# Doctors Are Inconsistent in Estimating Survival after CPR and Are Not Using Such Predictions Consistently in Determining DNACPR Decisions

**DOI:** 10.3390/geriatrics4020033

**Published:** 2019-05-03

**Authors:** Andrew C Kidd, Katie Honney, Lesley K Bowker, Allan B Clark, Phyo K Myint, Richard Holland

**Affiliations:** 1 Glasgow Pleural Disease Unit, Department of Respiratory Medicine, Queen Elizabeth University Hospital, Glasgow G51 4TF, UK; 2 Institute of Cancer Sciences, College of Medical, Veterinary and Life Sciences, University of Glasgow, Glasgow G12 8QQ, UK; 3 Older People’s Medicine, Norfolk and Norwich University Hospital, Norwich NR4 7UY, UK; katie.honney@nnuh.nhs.uk (K.H.); lesley.bowker@nnuh.nhs.uk (L.K.B.); 4 Norwich Medical School, Medicine and Health Sciences, University of East Anglia, Norwich NR4 7TJ, UK; allan.clark@uea.ac.uk; 5 Ageing Clinical and Experimental Research (ACER), Institute of Applied Health Sciences, School of Medicine, Medical Sciences & Nutrition, University of Aberdeen, Aberdeen AB25 2ZD, UK; phyo.myint@abdn.ac.uk; 6 Department of Medicine for the Elderly, Aberdeen Royal Infirmary, NHS Grampian AB25 2ZN, UK; 7 Leicester Medical School, College of Life Sciences, University of Leicester, Leicester LE1 7RH, UK; rch23@leicester.ac.uk

**Keywords:** DNACPR, futility, estimating survival, resuscitation

## Abstract

**Background**: It is unclear whether doctors base their resuscitation decisions solely on their perceived outcome. Through the use of theoretical scenarios, we aimed to examine the ‘do not attempt cardiopulmonary resuscitation’ (DNACPR) decision-making. **Methods**: A questionnaire survey was sent to consultants and specialty trainees across two Norfolk (UK) hospitals during December 2013. The survey included demographic questions and six clinical scenarios with varying prognosis. Participants were asked if they would resuscitate the patient or not. Identical scenarios were then shown in a different order and doctors were asked to quantify patients’ estimated chance of survival. **Results**: A total of 137 individuals (mean age 41 years (SD 7.9%)) responded. The response rate was 69%. Approximately 60% were consultants. We found considerable variation in clinician estimates of median chance of survival. In three out of six of our scenarios, the survival estimated varied from <1% to 95%. There was a statistically significant difference identified in the estimated median survival between those clinicians who would or would not resuscitate in four of the six scenarios presented. **Conclusion**: This study has highlighted the wide variation between clinicians in their estimates of likely survival and little concordance between clinicians over their resuscitation decisions. The diversity in clinician decision-making should be explored further.

## 1. Introduction

The published literature examining in-hospital cardiopulmonary resuscitation outcomes report markedly variable survival rates [1,2,3,4,5]. There are some patients for whom resuscitation in the event of cardiopulmonary arrest would be inappropriate and an increasingly extensive medical literature supports the selective use of cardiopulmonary resuscitation [6,7,8]. The concept of futility is widely discussed in the literature and described as uselessness or an “absence of any effect” [9]. Jecker concluded that when cardiopulmonary resuscitation is thought to be futile it is best explored from both quantitative and qualitative perspectives [10]. However, futility depends on the goals of treatment and is open to differing interpretations [11,12].

In the absence of a valid and relevant advance directive, in health care settings, it is required to make a provisional escalation plan in the case of deterioration and a ‘do not attempt cardiopulmonary resuscitation’ (DNACPR) order is often made as part of this management plan by doctors. The law regarding this in 2013 was the Mental Capacity Act 2005. This usually involves balancing the anticipated burdens (associated risks, complications and low survival) against the possible benefit (length and quality of life following cardiopulmonary resuscitation). Whilst such decisions are made with the best interest of each patient in mind, these can be subjective and potentially open to biases.

Despite a number of related guidelines and outcome studies, resuscitation decision-making by the medical profession has been the subject of very little research. It is unclear what influences actually determine a patient’s resuscitation status. The aim of this study was to explore whether doctors base their resuscitation decisions on their perceived outcome (i.e., a measure of futility) through the use of theoretical scenarios.

## 2. Materials and Methods

**Figure 1:**
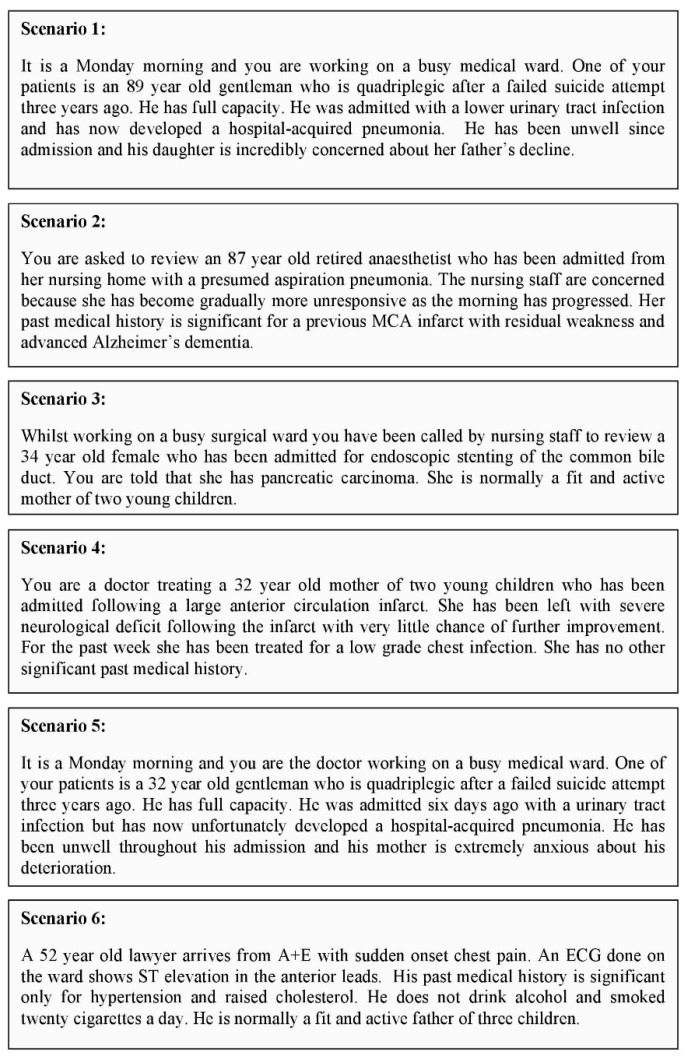
Scenarios sent to doctors of specialty trainee year three and above who frequently make ‘do not attempt cardiopulmonary resuscitation’ (DNACPR) decisions.

Doctors of specialty trainee year three and above who frequently make DNACPR decisions at the Norfolk and Norwich University Hospital (NNUH) and James Paget University Hospital (JPUH), Great Yarmouth were surveyed during December 2013. A link to the survey was distributed via Trust email accounts obtained from the respective human resources departments. The survey included demographic questions followed by six short resuscitation-based scenarios (developed from the participant feedback following a prior pilot study) in which the doctor completing the questionnaire was asked whether they would recommend resuscitation for the patient (Figure 1). The exact question was: “For each scenario state whether you would recommend resuscitation for the patient. Although we know you would usually have more data, including the views of patients and or relatives and any advanced directives about life sustaining treatment, for this survey we would like you to make your decision based only on the evidence presented”. Following completion of these questions, the doctor answering the questionnaire was shown the same scenarios in a different order and asked to quantify the estimated chance of survival to discharge (stratified as 0.1%, 1%, 2.5%, 5%, 7.5%, 10%, 20%, 30%, 40%, 50%, 60%, 70%, 80%, 90% and 100% on a non-linear scale) for the patient in each scenario.

### 2.1. Data Collection and Analysis

Descriptive statistics were tabulated (number (%) for categorical variables and median (range) for continuous variables). Estimated survival for each scenario was compared between those who would resuscitate and those who would not, using a Mann-Whitney test.

### 2.2. Ethical Approval

Ethical approval was obtained from the Research Ethics Committee of the Faculty of Medicine & Health Sciences, University of East Anglia (Reference: 2012/2013-60).

## 3. Results

A total of 232 surveys were sent and 162 individuals responded to the survey (69% response rate). Of these, a total of 137 respondents (mean age 41, SD 7.9) stated that they make do not attempt resuscitation decisions (Table 1). The majority were white British (67.2%); the remainder were Asian (21.3%), Other (8.2%), Black (2.5%) or Mixed Race (0.8%) Approximately 60% of respondents were consultants and three quarters had more than ten years of clinical experience. Representation by specialty was 45.9% medical, 22.6% surgical and 31.5% from other specialties.

We found significant variation in clinicians’ decision-making with regards to whether they would or would not resuscitate according to the different scenarios (Table 2). In scenario 6, we found that all clinicians who responded would attempt resuscitation (79.5%). However in scenario 4, we found significant variations in decision-making, with 39.4% and 40.8% choosing to resuscitate and not to resuscitate, respectively. We also found a sizeable number of clinicians who did not respond to the questions (18.2–21.2%).

**Figure 2:**
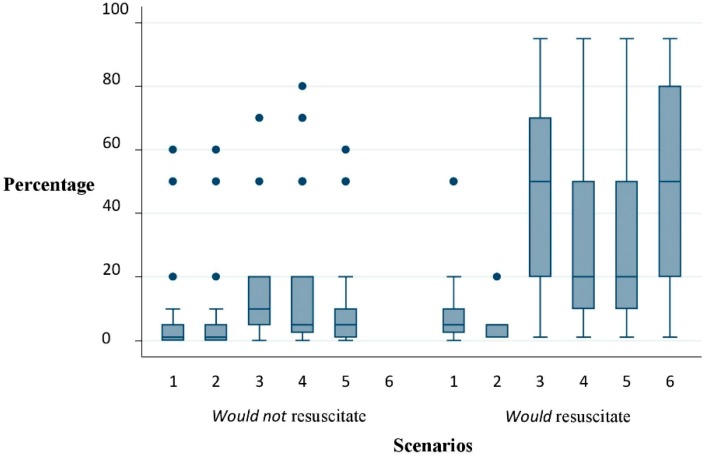
Boxplot showing the distribution of predicted survival by scenario and resuscitation decision.

We found considerable variation in clinicians’ estimates of median chance of survival (Table 3). For example, for scenarios 3 and 4 survival estimates ranged from <1% to 95%. There was a statistically significant difference identified in the estimated median survival between those clinicians who would or would not resuscitate, in four of the six scenarios presented. In addition, for those choosing not to resuscitate, the variation in estimates appeared to be somewhat less than for those who opted for resuscitation (Figure 2). Only in one case (scenario 6) was there complete agreement (i.e., 100% of the clinicians who responded to the question decided that the patient described should be resuscitated). However, even in this scenario there was wide variation in the estimated survival, ranging from 1 to 95% (interquartile range: 20–80, median 50%).

## 4. Discussion

The wide range of survival estimates, irrespective of whether or not resuscitation was chosen, suggests that there is little agreement between clinicians regarding survival and subsequently significant differences on decision to resuscitate or not. With the exception of scenario 6, all our scenarios generated opposing views about whether cardiopulmonary resuscitation (CPR) should be offered or a DNACPR order instituted.

There appeared to be a narrower range of estimated survival percentages for each scenario among those who would not resuscitate (excluding outliers). Most of these participants demonstrate that where they estimated survival of less than 20% or 10% that would generally justify a DNACPR order. In contrast, those who would resuscitate in the same scenario appeared to consider that even a 20% survival estimate was not ‘futile’ with regards to attempting resuscitation in the specific scenario. This appeared most notable where our scenarios included a young patient (32 years of age) where it is likely that other factors such as the presence of dependent children, perception of quality and value of life based on age or perception of patient views were influencing CPR choices.

Attempts to measure the futility of resuscitation based on the scoring of physiological and/or other prognostic features have resulted in limited success [13,14]. Pre-arrest scoring systems, such as the Good Outcome Following Attempted Resuscitation (GO-FAR) [15], Pre-Arrest Morbidity Index (PAM) [16] and the Prognosis After Resuscitation Score (PAR) [17] have been developed in an attempt to improve the prediction of outcomes following cardiopulmonary resuscitation. However, morbidity scores are likely to need further refinement in order to be a useful bedside tool for predicting success for individual patient resuscitation attempts.

Even if mortality prognostication scoring systems were adopted for use in predicting survival after cardiopulmonary arrest, they would clearly not be used as the sole tool used in making a final decision upon a patient’s resuscitation status. It is perhaps these ‘other’ influences, which are not easily captured in current research, that are most interesting to deliberate upon.

We have not explored how discussions with the patient and/or relatives influence DNACPR decision-making. Additional factors may include the functional status of the patient, their perceived quality of life, clinician’s personal experience and medico-legal aspects. The obvious challenge with accepting that other factors are at play is that patients are subject to the judgment of the clinician at the end of the bed as opposed to a uniform and generalizable decision that might be available if scoring systems for predicting outcome after cardiopulmonary arrest were widely utilized. This leaves clinicians open to accusations of overt bias (e.g., ageism) or unconscious bias. However, pushing clinicians to a more formulaic way of making important and emotive decisions and stopping them using their clinical judgment may not improve the quality of DNACPR orders.

Whilst our sample was diverse and drew across all sectors of hospital medical staff, the sample somewhat over-represented male hospital doctors (66% in our sample versus 55% nationally [18]) and somewhat under-represented black, Asian and minority ethnic (BAME) doctors (33% in our sample versus 41% nationally [18]).

### Strengths and Limitations

This is a reasonably large study in comparison to other studies in this field. However, the sample size is relatively small and larger studies are required. We sampled experienced doctors with a good response rate for surveys of this type. We believe that the self-selection bias was unlikely to have been a major problem as all scenarios generated a wide range of survival estimates, suggesting that no one belief set predominated. We deliberately created the scenarios in this study to be emotive in an attempt to represent the difficult reality of these decisions in clinical practice. This arguably limited our study in ascertaining the relationship between the estimated survival and decision to resuscitate, but also highlighted the fact that futility alone is not what makes a clinician’s overall decision. Furthermore, to ensure brevity and to maximize the response we did not ask for detailed explanations as to why or how participants reached their decisions. Another limitation was the lack of information regarding the respondent’s work settings, for example, whether they work in an ED department, geriatric ward, etc. A further limitation is that the terms “would resuscitate” and “would not resuscitate” are too simple. The variations in answers may be further clarified by having more specific discrete choices such as “do not start CPR in case of cardiopulmonary arrest” or “do not start or expand treatment with antibiotics, inotropes, mechanical ventilation, artificial administration of food, transfusion of blood/blood products, transfer to an intensive care unit and dialysis”. It is something to consider for future work. The scenarios also presented minimal physiological or pathological detail. While the scenarios did change around some variables and not others, it is difficult to draw conclusions about which variables were important. This may have resulted in the significant variation observed in the doctors’ options.

The data for this study was collected in 2013. In 2014 there was a significant change in the United Kingdom case law following the Tracey Judgement (R (David Tracey) vs. (1) Cambridge University Hospitals NHS Foundation Trust (2) Secretary of State for Health). This meant that all NHS Trusts have a legal duty to consult with and inform patients if a DNACPR order is placed on their medical records unless they have indicated that they do not wish to be involved or the discussion is likely to cause physical or psychological harm. Clinicians in the United Kingdom follow the DNACPR decision guidance issued by the General Medical Council (GMC) and work within the framework of the Mental Capacity Act 2005 (in England and Wales) and The Adults with Incapacity (Scotland) Act 2000. However, DNCAPR decision-making discussion with patients is often not possible, particularly in the geriatric setting. Ultimately in these circumstances it is a medical decision made by clinicians with patients’ ‘best interests’ in mind.

## 5. Conclusions

There are few studies that have examined resuscitation based decision-making in the medical profession. This study has highlighted the wide variation between clinicians in their estimates of the likely survival and little concordance between clinicians over resuscitation decisions. The diversity in clinician decision-making should be explored further through qualitative research in order to provide clinicians with education and guidance in informing these important decisions.

## Figures and Tables

**Table 1:** Characteristics of respondents.

	Mean/*n*	SD/%
Age	41.3	7.9
Male	81	66.4
Ethnicity		
White British	82	67.2
Asian	26	21.3
Other	10	8.2
Black	3	2.5
Mixed Race	1	0.8
Grade		
Consultant	70	58.3
SpR	32	26.7
Staff Grade	18	15
Experience (years)		
<5	26	21.9
5–10	5	4.2
>10	88	74.0
Specialty		
Other	26	21.7
Care of Elderly	21	17.5
Orthopaedic	15	12.5
Cardiology	10	8.3
Haematology	7	5.8
Urology	7	5.8
Gastroenterology	6	5
Endocrinology	5	4.2
Palliative	5	4.2
Colorectal	4	3.3
Respiratory	4	3.3
Neurology	3	2.5
Vascular	3	2.5
Renal	2	1.7
Upper GI	2	1.7

**Table 2:** Respondents who would and would not resuscitate by scenario.

Scenario	Respondents Who Would Not Resuscitate (%)	Respondents Who Would Resuscitate (%)	No Response (%)
1	82 (59.9)	30 (21.9)	25 (18.2)
2	105 (76.6)	7 (5.2)	25 (18.2)
3	11 (8.1)	100 (73.0)	26 (18.9)
4	54 (39.4)	56 (40.8)	27 (19.8)
5	42 (30.6)	66 (48.2)	29 (21.2)
6	0 (0)	109 (79.5)	28 (20.5)

**Table 3:** Resuscitation decision with median estimated survival range by scenario.

Scenario	Respondents Who Would Not Resuscitate Estimated Percentage Survival after CPR–Median (Range)	Respondents Who Would Resuscitate Estimated Percentage Survival after CPR–Median (Range)	*P*-Value *
1	1 (0.1–60)	5 (0.1–50)	0.001
2	1 (0.1–60)	1 (0.1–20)	0.130
3	10 (0.1–70)	50 (1–95)	0.005
4	5 (0.1–80)	20 (1–95)	<0.001
5	5 (0.1–60)	20 (1–95)	<0.001
6	-	50 (1–95)	-

* Mann-Whitney test comparing median survival.

## References

[1] Aarons E., Beeching N. (1991). Survey of “Do not resuscitate” orders in a district general hospital. BMJ.

[2] Schneider A.P., Nelson D.J., Brown D.D. (1993). In-hospital cardiopulmonary resuscitation: A 30-year review. J. Am. Board Fam. Pract..

[3] Tunstall-Pedoe H., Bailey L., Chamberlain D., Marsden A., Ward M., Zideman D. (1992). Survey of 3765 cardiopulmonary resuscitations in British hospitals (the BRESUS Study): Methods and overall results. BMJ.

[4] Peberdy M., Kaye W., Ornato J., Larkin G., Nadkarni V., Mancini M.E., Berg R., Nichol G., Lane-Trultt T. (2003). Cardiopulmonary resuscitation of adults in the hospital: A report of 14,720 cardiac arrests from the National Registry of Cardiopulmonary Resuscitation. Resuscitation.

[5] Sandroni C., Sandroni C., Nolan J., Cavallaro F., Antonelliet M. (2007). In-hospital cardiac arrest: Incidence, prognosis and possible measures to improve survival. Intensive Care Med..

[6] Hilberman M., Kutner J., Parsons D., Murphy D. (1997). Marginally effective medical care: Ethical analysis of issues in cardiopulmonary resuscitation (CPR). J. Med. Ethics.

[7] (2016). Guidance from the British Medical Association, the Resuscitation Council (UK) and the Royal College of Nursing. Decisions Relating to Cardiopulmonary Resuscitation (Previously Known as the ‘Joint Statement’).

[8] Williams R. (1993). The do-not-resuscitate decision: Guidelines for policy in the adult. J. Royal Coll. Phys. Lond..

[9] Ardagh M. (2000). Futility has no utility in resuscitation medicine. J. Med. Ethics.

[10] Jecker N. (2007). Medical futility: A paradigm analysis. HEC Forum.

[11] Weijer C., Elliott C. (1995). Pulling the plug on futility. BMJ.

[12] Bruce-Jones P. (1996). Resuscitation decisions in the elderly: A discussion of current thinking. J. Med. Ethics.

[13] Glance L.G., Osler T., Shinozaki T. (1998). Intensive care scoring systems to predict death: A cost effective analysis. Crit. Care Med..

[14] Rodriquez R.M., Wang N.E., Pearl R.G. (1997). Prediction of poor outcome of intensive care unit patients admitted from the emergency department. Crit. Care Med..

[15] Ebell M.H., Jang W., Shen Y., Geocadin R.G., for the Get With the Guidelines–Resuscitation Investigators (2013). Development and Validation of the Good Outcome Following Attempted Resuscitation (GO-FAR) Score to Predict Neurologically Intact Survival After In-Hospital Cardiopulmonary Resuscitation. JAMA Intern. Med..

[16] George A.L., Folk B.P., Crecelius P.L., Campbell W.B. (1989). Pre-arrest morbidity and other correlates of survival after in-hospital cardiopulmonary arrest. Am. J. Med..

[17] O’Keeffe S., Ebell M.H. (1994). Prediction of failure to survive following in-hospital cardiopulmonary resuscitation: Comparison of two predictive instruments. Resuscitation.

[18] Rimmer A. (2016). Ethnic diversity in NHS trusts. BMJ.

